# Application of DEN refinement and automated model building to a difficult case of molecular-replacement phasing: the structure of a putative succinyl-diaminopimelate desuccinylase from *Corynebacterium glutamicum*

**DOI:** 10.1107/S090744491104978X

**Published:** 2012-03-16

**Authors:** Axel T. Brunger, Debanu Das, Ashley M. Deacon, Joanna Grant, Thomas C. Terwilliger, Randy J. Read, Paul D. Adams, Michael Levitt, Gunnar F. Schröder

**Affiliations:** a Departments of Molecular and Cellular Physiology, Neurology and Neurological Sciences, and Photon Science, Stanford University, USA; b Howard Hughes Medical Institute, USA; c Joint Center for Structural Genomics, USA; d Stanford Synchrotron Radiation Lightsource, SLAC National Accelerator Laboratory, USA; e Protein Sciences Department, Genomics Institute of the Novartis Research Foundation, USA; f Los Alamos National Laboratory, USA; g Department of Haematology, University of Cambridge, Cambridge Institute for Medical Research, England; h Department of Bioengineering, University of California at Berkeley and Lawrence Berkeley National Laboratory, Berkeley, USA; i Department of Structural Biology, Stanford University School of Medicine, USA; j Institute of Complex Systems (ICS-6), Forschungszentrum Jülich, Germany

**Keywords:** reciprocal-space refinement, DEN refinement, real-space refinement, automated model building, succinyl-diaminopimelate desuccinylase

## Abstract

Phasing by molecular replacement remains difficult for targets that are far from the search model or in situations where the crystal diffracts only weakly or to low resolution. Here, the process of determining and refining the structure of Cgl1109, a putative succinyl-diaminopimelate desuccinylase from *Corynebacterium glutamicum*, at ∼3 Å resolution is described using a combination of homology modeling with *MODELLER*, molecular-replacement phasing with *Phaser*, deformable elastic network (DEN) refinement and automated model building using *AutoBuild* in a semi-automated fashion, followed by final refinement cycles with *phenix.refine* and *Coot*. This difficult molecular-replacement case illustrates the power of including DEN restraints derived from a starting model to guide the movements of the model during refinement. The resulting improved model phases provide better starting points for automated model building and produce more significant difference peaks in anomalous difference Fourier maps to locate anomalous scatterers than does standard refinement. This example also illustrates a current limitation of automated procedures that require manual adjustment of local sequence misalignments between the homology model and the target sequence.

## Abstract 2

DEN refinement and automated model building with *AutoBuild* were used to determine the structure of a putative succinyl-diaminopimelate desuccinylase from *C. glutamicum*. This difficult case of molecular-replacement phasing shows that the synergism between DEN refinement and *AutoBuild* outperforms standard refinement protocols.

## 1. Introduction

Successful molecular-replacement phasing depends on a number of factors such as the proximity of the search model to the true structure, the quality and completeness of the diffraction data (especially at lower resolution), the solvent content, the presence of noncrystallographic symmetry and the limiting resolution (*d*_min_) of the crystals. Although recent advances in reciprocal-space refinement such as deformable elastic network (DEN) refinement (Schröder *et al.*, 2010 ▶), jelly-body refinement (Murshudov *et al.*, 2011 ▶) and real-space refinement (DiMaio *et al.*, 2011 ▶) enable structure determination from more distant models, the ultimate success of molecular replacement phasing depends on whether previously unknown parts of the model become visible in the electron-density maps or whether conformational changes in the structure are uniquely determined.

DEN refinement consists of torsion-angle refinement interspersed with *B*-factor refinement in the presence of a sparse set of distance restraints (typically one per atom, randomly selected) which are initially obtained from a reference model (Schröder *et al.*, 2010 ▶). The reference model can simply be the starting model for refinement or it can be a homology or predicted model that provides external information. During the process of torsion-angle refinement with a slow-cooling simulated-annealing schema, the DEN distance restraints are adjusted in order to fit the diffraction data. The degree of this adjustment or deformation of the initial distance restraints is controlled by a parameter γ. The method of jelly-body refinement (Murshudov *et al.*, 2011 ▶) bears some resemblance to the special case of DEN refinement with γ = 1. The weight of the DEN distance restraints is controlled by another parameter, *w*_DEN_. A two-dimensional grid search for (γ, *w*_DEN_) is performed in which multiple refinements for each parameter pair are performed with different initial random-number seeds for the velocity assignments of the torsion-angle molecular-dynamics method and different randomly selected DEN distance restraints. The globally optimal model (in terms of minimal *R*_free_, possibly assisted by geometric validation criteria) is then used for further refinement and model building. By default, the last two macrocycles of the DEN refinement protocol are performed without any DEN restraints, so the resulting model is not strained or biased by the reference model (although such restraints can be useful at very low resolution). In other words, the DEN restraints guide the refinement path, increasing the chances of obtaining a better model than with standard refinement. In addition, the deformability of the DEN restraints makes this method more general than rigid-body or normal-mode refinement. Thus, DEN refinement is a general refinement method that can be applied to any starting model and reference model. In practice, the reference model is likely to be identical to the starting model. However, there are situations in which the reference model can be different from the starting model. For example, re-refinements of existing structures can be performed using structures of homologous proteins that were not available at the time the original structure was determined.

A number of highly automated procedures for model building and model rebuilding have recently been developed (Levitt, 2001 ▶; Oldfield, 2002 ▶, 2003 ▶; Ioerger & Sacchettini, 2003 ▶; DePristo *et al.*, 2005 ▶; Cowtan, 2006 ▶; Langer *et al.*, 2008 ▶; Terwilliger *et al.*, 2008 ▶). A key feature of several of these procedures is alternation between model building and calculation of electron-density maps. Each local improvement in the model leads to an overall improvement in the map, which in turn makes additional improvements in the model possible. In this work, we use one of these procedures, the *AutoBuild* method (Terwilliger *et al.*, 2008 ▶) as implemented in *PHENIX *(Adams *et al.*, 2010 ▶), as a core tool for model improvement. In one cycle of model rebuilding with *AutoBuild*, a density-modified electron-density map is calculated beginning with phases from the working model and including any available experimental phase information. A new model is then built and refined with *phenix.refine* (Afonine *et al.*, 2005 ▶). Two methods for rebuilding the working model are used here. In the first method, several new models (or segments) are built without reference to the working model. The parts of the new models and the working model that best fit the electron-density map are then merged together to form a composite model. Using this procedure, the model can change in any way during rebuilding. In the second method, termed ‘rebuilding in place’, segments of the working model are rebuilt one at a time, maintaining connectivity and sequence alignment. This ‘rebuilding-in-place’ procedure therefore adjusts the position of existing atoms in the structure and can be thought of as an extension of refinement.

In this paper, we describe the process of determining the crystal structure of Cgl1109 (Joint Center for Structural Genomics target 376512 listed in TargetDB; http://targetdb.sbkb.org/TargetDB/), a putative succinyl-diamino­pimelate desuccinylase from *Corynebacterium glutamicum*, using a combination of molecular-replacement phasing, refinement and semi-automated model building. At the later stages, experimental phase information from SeMet MAD phasing was included in the refinement. It should be noted that these MAD phases were of insufficient quality to allow automated model building, and manual building would have been exceedingly difficult and time-consuming even for a highly skilled crystallographer (see §[Sec sec3.6]3.6). Thus, molecular-replacement phasing was attempted. However, manual interpretation of the initial electron-density map again proved difficult. Indeed, Cgl1109 was one of the cases used to test the performance of real-space refinement of the molecular-replacement solution in conjunction with the *Rosetta* empirical energy function (DiMaio *et al.*, 2011 ▶; case 10 in Table 1 in this reference), but the refinement was not completed owing to poor or disordered density in numerous regions and low resolution (*R*_free_ = 0.39; Table 1 in DiMaio *et al.*, 2011 ▶).

Here, we present an independent structure determination of Cgl1109 at ∼3 Å resolution without use of the previous *Rosetta* model and molecular-replacement solution. A homology model of Cgl1109 was created using sequence alignment with *PROMALS*3*D* (Pei *et al.*, 2008 ▶) and modeling with *MODELLER* (Sali & Blundell, 1993 ▶) starting from the structure of succinyl-diaminopimelate desuccinylase from the β-proteobacterium *Neisseria meningitidis* (PDB entry 1vgy; Badger *et al.*, 2005 ▶). The structure was determined by molecular replacement with *Phaser* (McCoy *et al.*, 2007 ▶) using a model edited with *Sculptor* (Bunkóczi & Read, 2011 ▶), followed by DEN refinement with a full (γ, *w*_DEN_) grid search (Schröder *et al.*, 2010 ▶), automated model building with *AutoBuild*, determination of the selenium sites by anomalous difference Fourier maps, calculation of MAD phase probability distributions using a maximum-likelihood method (Burling *et al.*, 1996 ▶) and completion of the refinement in a semi-automated fashion using *AutoBuild* and *phenix.refine* (Adams *et al.*, 2010 ▶) with the MLHL target function (Pannu *et al.*, 1998 ▶). The final model has excellent geometry and *R*_cryst_ and *R*_free_ values of 0.238 and 0.257, respectively, at 2.97 Å resolution.

This example shows that DEN refinement with a full (γ, *w*_DEN_) grid search generally produces models that are closer to the true structure than standard (gradient-descent) or simulated-annealing refinement methods, resulting in improved model phases and better *R* values. The improved model phases in turn provide better starting points for automated model building with *AutoBuild*. This approach ultimately produced a well refined structure that would have been very difficult to achieve with manual model building and standard refinement. Moreover, the improved model phases produce more significant difference peaks that better locate the anomalous diffraction selenium sites. Compared with the *Rosetta* refinement method (DiMaio *et al.*, 2011 ▶), DEN refinement has the advantage that it does not require extensive empirical energy-function simulations and that it has been shown to also work well for structures determined at low resolution (worse than 3.5 Å). The successful application to Cgl1109 demonstrates that DEN refinement also has significant utility for structures determined at ∼3 Å resolution, especially for cases of anisotropic diffraction and/or high *B* factors. The research performed in this paper also serves as a tutorial for the combined use of various methods and computer software systems to tackle difficult molecular-replacement cases. The corresponding data files have been made available on the *CNS* website in the tutorial section for DEN refinement.

## 2. Materials and methods

### 2.1. Crystallization

Cgl1109 was expressed, purified and crystallized using the JCSG high-throughput structural biology pipeline (Elsliger *et al.*, 2010 ▶) and standard JCSG protocols with crystallization modifications. Briefly, clones were generated using the Polymerase Incomplete Primer Extension (PIPE) cloning method (Klock *et al.*, 2008 ▶). The gene encoding Cgl1109 (GenBank NP_600337, gi|19552335; UniProt Q59284) was PCR-amplified from *C. glutamicum* 534 genomic DNA using *PfuTurbo* DNA polymerase (Stratagene) and I-PIPE primers (forward primer, 5′-ctgtacttccagggcCTGTACTTCCAGGGCATGAACTCTG­AACTCAAACCAGGATTAG-3′; reverse primer, 5′-aattaa­gtcgcgttaAATTAAGTCGCGTTACTCGCTCAGGTACTG­CTTCAAAATTGC-3′; target sequence in upper case) that included sequences for the predicted 5′ and 3′ ends. The genomic DNA used here and obtained from the American Type Culture Collection (ATCC) contained two amino-acid substitutions (Glu4Asn and Lys6Gln) and one amino-acid deletion (Leu5), as confirmed by DNA sequencing, when compared with the available GenBank sequence from *C. glutamicum* 534; these mutations are unlikely to affect the biochemical properties of the enzyme based on their locations. Expression was performed in selenomethionine-containing medium at 298 K. Selenomethionine was incorporated *via* inhibition of methionine biosynthesis (Van Duyne *et al.*, 1993 ▶), which does not require a methionine-auxotrophic strain. The protein was purified by two steps of nickel-chelating chromatography (GE Healthcare) with an intermediate step involving TEV protease cleavage of the purification tag and was concentrated to 18.5 mg ml^−1^ by centrifugal ultrafiltration (Millipore) for crystallization trials. Crystals used for structure determination were grown using Microseed Matrix Screening (MMS; Ireton & Stoddard, 2004 ▶; D’Arcy *et al.*, 2007 ▶) as implemented with an Oryx8 crystallization robot (Douglas Instruments). Initial seed crystals used for MMS were grown using the nanodroplet vapor-diffusion method from sitting drops composed of 200 nl protein solution mixed with 200 nl crystallization solution equilibrated against a 50 µl reservoir at 293 K for 48 days prior to harvest. The crystals used for the seed stock were obtained using a precipitating reagent consisting of 0.2 *M* MgCl_2_, 30% PEG 400, 0.1 *M* HEPES pH 7.5. The entire crystallization drop (400 nl) containing the seed crystals was aspirated using a pipette and placed in a Seed Bead tube (Hampton Research) stored on ice. To ensure that all crystals were transferred to the Seed Bead tube, the empty shelf was rinsed with 50 µl mother liquor. The Seed Bead tube con­taining the seed stock was vortexed vigorously for three intervals of 30 s, keeping the tube on ice between each vortex. Final MMS crystallization plates were set up on the Oryx8 as sitting drops composed of 150 nl protein, 100 nl crystallization solution and 50 nl seed stock. The final crystals used for structure determination were obtained from a crystallization reagent consisting of 43.1% polyethylene glycol 400, 0.2 *M* sodium chloride, 0.1 *M* sodium/potassium phosphate pH 6.41 at 293 K for 21 d prior to harvest. 6 m*M* ZnCl_2_ was added to the protein prior to setup. No additional cryoprotectant was added to the crystal. Initial screening for diffraction was carried out using the Stanford Automated Mounting system (SAM; Cohen *et al.*, 2002 ▶) at the Stanford Synchrotron Radiation Lightsource (SSRL; Menlo Park, California, USA).

### 2.2. X-ray data collection, processing, structure validation and deposition

MAD data were collected on beamline 9-2 at the SSRL at wavelengths corresponding to the high-energy remote (λ_1_), inflection point (λ_2_) and peak (λ_3_) wavelengths of a selenium MAD experiment using the *Blu-Ice* (McPhillips *et al.*, 2002 ▶) data-collection environment. The data sets were collected at 100 K using a MAR Mosaic 325 CCD detector (Rayonix, USA). The MAD data were integrated and reduced using *XDS* (Kabsch, 2010 ▶) and scaled with *XSCALE* (Kabsch, 2010 ▶). Diffraction data and refinement statistics are summarized in Table 1 ▶. The quality of the crystal structure was analyzed using the JCSG Quality Control server (http://smb.slac.stanford.edu/jcsg/QC), which verifies the stereochemical quality of the model using *AutoDepInputTool* (Yang *et al.*, 2004 ▶), *MolProbity* (Chen *et al.*, 2010 ▶) and *WHAT IF* v.5.0 (Vriend, 1990 ▶); the agreement between the atomic model and the data using *SFCHECK* v.4.0 (Vaguine *et al.*, 1999 ▶) and *RESOLVE* (Terwilliger, 2000 ▶); the protein sequence using *ClustalW* (Thompson *et al.*, 1994 ▶); atom occupancies using *MOLEMAN*2 (Kleywegt, 2000 ▶) and the consistency of NCS pairs; and evaluates *R*_free_/*R*_cryst_ and the maximum/minimum *B* factors. Atomic coordinates and experimental data for Cgl1109 from *C. glutamicum* to 2.97 Å resolution (PDB entry 3tx8) have been deposited in the Protein Data Bank (http://www.wwpdb.org).

### 2.3. Homology modeling, structure determination and refinement

*PROMALS*3*D* (Pei *et al.*, 2008 ▶) was used for primary-sequence alignment, *MODELLER* (Sali & Blundell, 1993 ▶) was used for profile generation and homology modeling, *Sculptor* (Bunkóczi & Read, 2011 ▶) and *Phaser* (McCoy *et al.*, 2007 ▶) were used for molecular-replacement phasing, *CNS* v.1.3 was used for DEN refinement (Schröder *et al.*, 2010 ▶), *AutoBuild* (Terwilliger *et al.*, 2008 ▶) was used for automated model building, *Coot* (Emsley *et al.*, 2010 ▶) was used for manual rebuilding and structure validation, *CNS* was used for MAD phasing and density modification (Brünger *et al.*, 1998 ▶), *phenix.refine* (Adams *et al.*, 2010 ▶) was used for final refinement cycles and *PyMOL* (DeLano, 2002 ▶) was used for molecular illustrations and structure and electron-density map superposition.

## 3. Results and discussion

### 3.1. Search for similar structures, primary-sequence alignment and homology modeling

**Figure 1:**
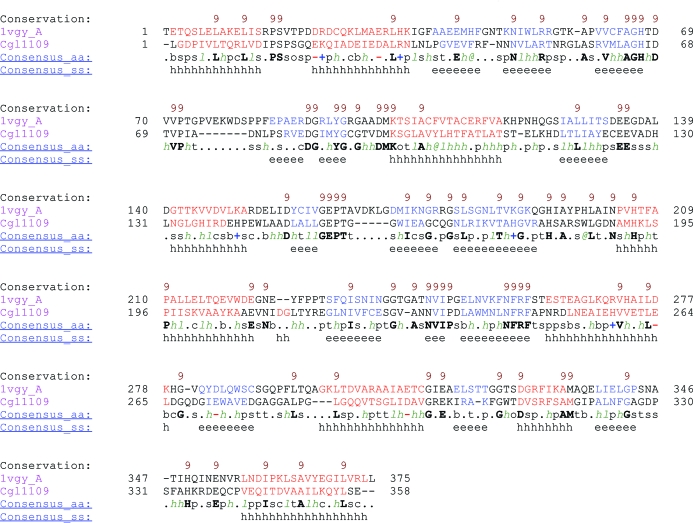
Primary-sequence alignment between 1vgy (chain *A*) and Cgl1109. The alignment obtained by *PROMALS*3*D* (Pei *et al.*, 2008 ▶) is shown. The first line in each block shows conservation indices for positions with a conservation index above 4. The last two lines show consensus amino-acid sequence (Consensus_aa) and consensus predicted secondary structure (Consensus_ss). The representative sequences are named in magenta and are colored according to predicted secondary structure (red, α-helix; blue, β-strand). The first and last residue numbers of each sequence in each alignment block are shown before and after the sequences, respectively. Consensus-predicted secondary-structure symbols: α-helix, h; β-strand, e. Consensus amino-acid symbols are as follows (conserved amino acids are shown in bold uppercase letters); aliphatic (I, V, L), *l*; aromatic (Y, H, W, F), @; hydrophobic (W, F, Y, M, L, I, V, A, C, T, H), *h*; alcohol (S, T), o; polar residues (D, E, H, K, N, Q, R, S, T), p; tiny (A, G, C, S), t; small (A, G, C, S, V, N, D, T, P), s; bulky residues (E, F, I, K, L, M, Q, R, W, Y), b; positively charged (K, R, H), +; negatively charged (D, E), −; charged (D, E, K, R, H), c. Note that the sequence numbers refer to the genomic sequence of Cgl1109 (taking into account the minor mutations in the construct used for crystallization; see text) and 1vgy. The residue numbering in the deposited PDB file (PDB entry 3tx8) begins with the first residue of the expression construct used, so it is offset by 11 residues compared with the genomic sequence.

A profile of structures related to the genomic sequence of Cgl1109 (Fig. 1 ▶) was generated using the *MODELLER* build_profile.py script (http://www.salilab.org/modeller/tutorial/basic.html) and the current protein database file pdb_95.pir (updated 24 February 2011) available in the supplementary file download section of the *MODELLER* website. This produced a list of eight homologous structures (PDB entries 1cg2, 3ct9, 2f7v, 3gb0, 3isz, 3pfo, 2rb7 and 1vgy) with sequence identities that varied between 24 and 28%. A cluster analysis of these structures using the *MODELLER* compare.py script revealed that they are all relatively equidistant from each other, with the exception of PDB entries 3isz and 1vgy, which are closer to each other than to the other structures. Since there is no significant difference in terms of sequence identity to the target structure among these candidate models, the one with the highest resolution and best *R*_free_ value was chosen for all further calculations (PDB entry 1vgy chain *A*, referred to as 1vgy-*A* in the following), which was also the template used for *Rosetta*-based molecular replacement (DiMaio *et al.*, 2011 ▶).

The success of molecular replacement depends on optimal sequence alignment between homologous structure and target sequence (Schwarzenbacher *et al.*, 2004 ▶; Bunkóczi & Read, 2011 ▶). To make some use of the structural information in the primary-sequence alignment we used the *PROMALS*3*D* program (Pei *et al.*, 2008 ▶), resulting in the alignment shown in Fig. 1 ▶. *PROMALS*3*D* can produce more accurate sequence alignments compared with methods that do not make use of secondary-structure information for sequence pairs with at least 20% identity (Pei *et al.*, 2008 ▶). Other methods such as *HHpred* (Söding, 2005 ▶) that include secondary-structure information may provide alternative alignments (see §[Sec sec4]4).

The primary-sequence alignment obtained with *PROMALS*3*D* and the structure of 1vgy-*A* were used as input for the generation of a homology model using the model-single.py script of *MODELLER*. All default parameters were used except that the a.very_fast() option was specified to perform a limited amount of target-function optimization with conjugate-gradient minimization. This limited amount of energy minimization keeps the resulting homology model closer to the crystal structure of 1vgy-*A*, which may be a benefit since 1vgy-*A* itself produces a molecular-replacement solution (see §[Sec sec3.2]3.2). In general, it might be beneficial to try this fast optimization method as well as models generated by *MODELLER* with more extensive optimization and then to judge the models according to the molecular-replacement score.

### 3.2. Molecular-replacement phasing

Molecular-replacement phasing using *Phaser* (McCoy *et al.*, 2007 ▶) was performed with two different search models: the 1vgy-*A* crystal structure and the homology model of Cgl1109 obtained by *MODELLER*. The original *B* factors were used for the 1vgy-*A* search model. The diffraction data for the Cgl1109 crystal structure were quite anisotropic and the effective overall *B* factors along the principal axes of the unit cell ranged from 60 to 110 Å^2^. The relatively high anisotropy and high *B* factors made the structure determination considerably more challenging than for many other structures at a similar resolution of about 3 Å. After clustering of the rotation-function and translation-function peaks and the purging of peaks below a 75% threshold (the default settings in *Phaser*), a single solution emerged with RFZ = 3.2, TFZ = 9.7, LLG = 65, *R*_cryst_ = 0.65 and six clashes.

The *MODELLER* search model (with *B* factors set to a uniform value of 50 Å^2^) was first edited using *Sculptor* (Bunkóczi & Read, 2011 ▶) with the *PROMALS*3*D* alignment (Fig. 1 ▶) in order to trim surface side chains (as suggested by Schwarzenbacher *et al.*, 2004 ▶) and to modify the *B* factors of the search model according to sequence similarity between Cgl1109 and 1vgy-*A* (the similarity score was used for the *B*-­factor modeling and the Schwarzenbacher score was used for the pruning). After clustering of the rotation-function and translation-function peaks and purging peaks below a 75% threshold (default settings in *Phaser*), a single solution emerged with RFZ = 3.2, TFZ = 9.9, LLG = 75, *R*_cryst_ = 0.65 and 11 clashes. The position and orientation of this solution was very similar to that obtained with molecular replacement using the 1vgy-*A* search model, lending credence to the correctness of the solution. Furthermore, the solution was determined to be identical to that found by molecular replace­ment with the *Rosetta* search model (DiMaio *et al.*, 2011 ▶) apart from application of symmetry and lattice operators. However, *Phaser* was unable to produce the correct solution when using a fully optimized model obtained with the default settings in *MODELLER* [as opposed to the minimal a.very_fast() setting]; inspection of the optimized *MODELLER* model revealed that it had significantly moved away from the 1vgy-*A* template and thus was apparently too distant from the true structure of Cgl1109 to produce a molecular-replacement solution. This example shows that it is useful to try different homology models and to score them according to the criteria provided by the particular molecular-replacement method used, *e.g.* rotation-function and translation-function *Z* scores and log-likelihood gain in *Phaser*. In general, it is advisable to try additional searches in which the search model is broken up into subdomains that may exhibit different relative orientations and translations. However, this was unnecessary for Cgl1109 as the subdomain placements were very similar between Cgl1109 and 1vgy-*A* (see below).

**Figure 2:**
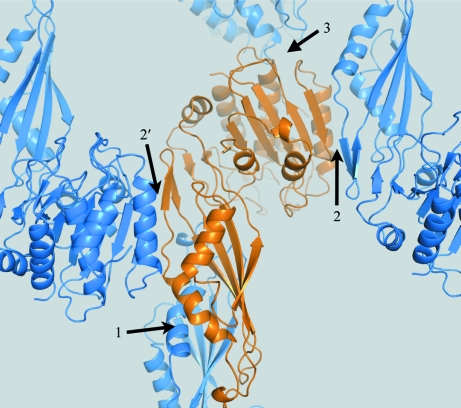
Interaction between symmetry-related molecules. A primary molecule (orange) and the nearest symmetry-related molecules (blue) obtained by applying the symmetry operators of the space group of the crystal (*P*6_5_22) to the primary molecule are shown, as well as lattice translations. Taken together, all these molecules form a network of interactions which is connected throughout the crystal in all three dimensions. The molecules interact through three interfaces, labelled 1, 2 and 3. Interface 2′ is related by crystallographic symmetry to interface 2. Of the three interfaces, interface 1 involves the most extensive interactions, with a buried suface area of 1569 Å^2^ (compared with 541 Å^2^ for interface 2 and 276 Å^2^ for interface 3; the buried surface areas were computed with the *PDBePISA* server). Considering the extensive interactions, interface 1 is likely to promote dimerization of the molecule, as is also suggested by the *PDBePISA* server.

A further validation of a molecular-replacement solution is provided by the overall crystal-packing arrangement and connectivity of the arrangement, *i.e.* no empty spaces should be left between the layers of molecules. Fig. 2 ▶ illustrates the connectivity of the arrangement and the three different interfaces that are created by symmetry and lattice operators.

### 3.3. DEN refinement

DEN refinement generally requires a starting model that matches the primary sequence of the target structure. Therefore, the molecular-replacement solution obtained from the minimally optimized *MODELLER* model was used as the starting point for DEN refinement. Side chains that were pruned by *Sculptor* were added back to the model by superimposing the complete model obtained by *MODELLER* on the *Phaser* molecular-replacement solution. All *B* factors were reset to a uniform value (50 Å^2^). The resulting coordinates were used as both the starting and reference model for DEN refinement (Schröder *et al.*, 2010 ▶). The refinement protocol was similar to that used in previous work (Schröder *et al.*, 2010 ▶; as also described in the tutorial for DEN refinement in *CNS* v.1.3; http://cns-online.org/v1.3/) except that isotropic restrained individual *B*-factor refinement was carried out instead of restrained group *B*-factor refinement as appropriate for the resolution of Cgl1109. Specifically, ten macrocycles of torsion-angle refinement and restrained individual *B*-factor refinement were performed in which the first cycle always used γ = 0, the following seven cycles used a specified value for γ (see below) and the last two cycles were performed without DEN restraints. The MLF target function (Pannu & Read, 1996 ▶) was used for the refinement against the diffraction data at the inflection point (the same diffraction data that were used in the work by DiMaio *et al.*, 2011 ▶). In the final stages of refinement, the diffraction data at the high-energy remote wavelength were used (see below).

DEN distance restraints were generated from *N* randomly selected pairs of atoms in the reference model that were separated by not more than ten residues along the polypeptide sequence and were separated by 3–15 Å in space (default settings for DEN refinement in *CNS*). The value of *N* was chosen to be equal to the number of atoms, so the set of distance restraints was relatively sparse, with an average of one restraint per atom.

**Figure 3:**
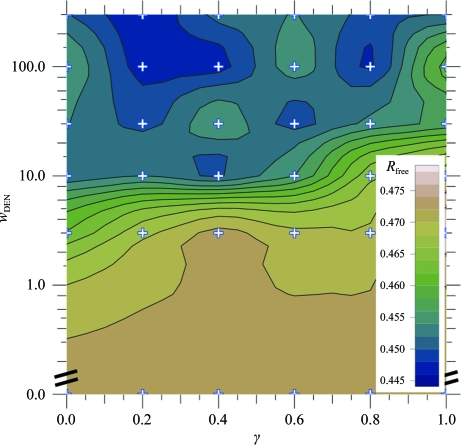
DEN refinement starting from molecular-replacement solution. The best *R*_free_ value for each parameter pair (γ, *w*_DEN_) among 20 repeats is shown; for each parameter pair we performed 20 repeats of the DEN-refinement protocol consisting of ten macrocycles of torsion-angle refinement and restrained individual *B*-factor refinement (for details, see text). The *R*_free_ value is contoured using values calculated on a 6 × 6 grid (marked by small + signs) where the parameter γ is (0.0, 0.2, 0.4, 0.6, 0.8, 1.0) and *w*_DEN_ is (0, 3, 10, 30, 100, 300); the results for *w*_DEN_ = 0 (*i.e.* torsion-angle refinement without DEN restraints) are independent of γ and the same value was used for all grid points with *w*_DEN_ = 0. The value of *R*_free_ varies from 0.444 to 0.479. The contour plot shows two pronounced minima in the range 300 ≥ *w*_DEN_ ≥ 100, with the absolute minimum at *w*_DEN_ = 300, γ = 0.2.

**Figure 4:**
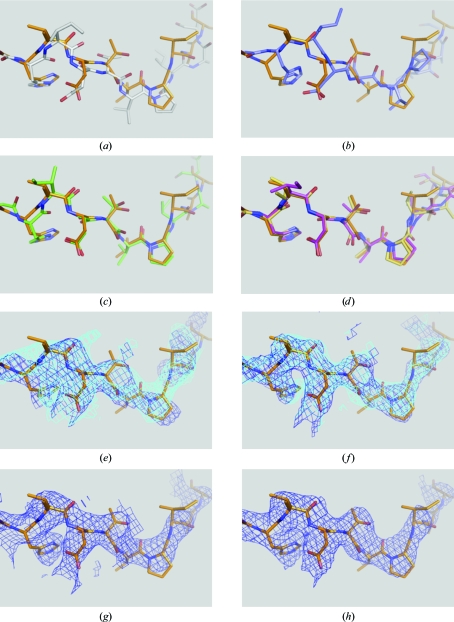
Comparison of various refinements and maps for residues 66–77. The sequence numbers refer to the genomic sequence of Cgl1109 (see Fig. 1 ▶). (*a*) Standard refinement (gray) *versus* the final model (orange). (*b*) Standard refinement and one round of *AutoBuild* (blue) *versus* the final model (orange). (*c*) DEN refinement (green) *versus* the final model (orange sticks). (*d*) DEN refinement and one round of *AutoBuild* (magenta) *versus* the result of semi-automated rebuilding (yellow) *versus* the the final model (orange). (*e*) 2*mF*_o_ − *DF*_c_ electron-density map after standard refinement (blue mesh) and a subsequent round of *AutoBuild* (cyan mesh) *versus* the final structure (orange sticks). (*f*) 2*mF*_o_ − *DF*_c_ electron-density map after DEN refinement (blue) and a subsequent round of *AutoBuild* (cyan) *versus* the final structure (orange sticks). (*g*) Electron-density map obtained by density modification of the MAD map (blue) *versus* the final structure (orange sticks). (*h*) 2*mF*_o_ − *DF*_c_ electron-density map (blue mesh) of the final model (orange sticks).

We determined the optimum values of the γ and *w*_DEN_ parameters of DEN refinement by a global two-dimensional grid search (Fig. 3 ▶). At each grid point, 20 refinement repeats were performed with different random initial velocities and different randomly selected DEN distances. We used 30 combinations of six γ values (0.0, 0.2, 0.4, 0.6, 0.8 and 1.0) and five *w*_DEN_ values (3, 10, 30, 100 and 300); we also included 20 repeats with *w*_DEN_ = 0 (corresponding to using the refinement protocol without DEN restraints, with the results being independent of γ). Of all the resulting models, the one with the lowest *R*_free_ value (0.444; Fig. 3 ▶ and Table 1 ▶) was used for subsequent model building and refinement. Generally, if there are multiple models with similar low *R*_free_ values, one could choose the one with the better geometry. The resulting model was substantially better in many places than what could be achieved using a standard refinement protocol (for a representative example, compare Figs. 4 ▶ *a* and 4 ▶ *b* and see below).

### 3.4. First round of automated model building with *AutoBuild*

Starting from the best DEN-refined structure, automated model building with *AutoBuild* (Terwilliger *et al.*, 2008 ▶) was performed. The default settings for rebuilding the model without the addition or deletion of residues (the rebuild_in_place=true option in *AutoBuild*) were used except that ‘morphing’ was enabled and the resolution for multiple model building was set to the limiting resolution of the diffraction data at the inflection-point wavelength (3.17 Å). The morphing process in *AutoBuild* consists of identifying a coordinate shift to apply to each backbone N atom that maximizes the local density correlation between the model and the map (Terwilliger *et al.*, submitted). These coordinate shifts are smoothed and applied to the structure to generate a morphed structure. An initial map was used for *AutoBuild* consisting of the average of the 2*mF*_o_ − *DF*_c_ electron-density maps corresponding to the top 20 models (in terms of *R*_free_) obtained from DEN refinement. Such map averaging can be beneficial (Rice *et al.*, 1998 ▶), although in this particular case using the average map was similar to using the map obtained from the top solution. This round of automatic model building produced further improvements in the model (Figs. 4 ▶ *d* and 5 ▶ *d*) and lowered the *R* values (*R*_free_ = 0.418, *R*_cryst_ = 0.327; Table 2 ▶).

At this point, it became clear from the electron-density maps produced by DEN refinement (2*mF*_o_ − *DF*_c_ map) and *AutoBuild* (both 2*mF*_o_ − *DF*_c_ and density-modified maps) that the model contained several incorrect sequence registers, resulting in distorted α-helices and bulging loops that had no electron density associated with them (a striking example is shown in Fig. 6 ▶). DEN refinement and *AutoBuild* are currently unable to automatically correct such sequence-register shifts and deformed α-helices. In particular, *AutoBuild* has no facility for automatic adjustment of sequence register or missing residues when building with the rebuild_in_place approach. Still, it was possible to correct these errors by semi-automated rebuilding and manual model building as outlined below. In principle, completely automated rebuilding of the model can be performed for structures at 3 Å resolution (*e.g.* starting from an experimental electron-density map or a density modified map of a molecular-replacement solution), but for Cgl1109 this approach was not successful, presumably owing to the relatively high anisotropy and *B* values of the crystal structure. It should be noted that no experimental MAD phase information had been used up to this stage of the refinement process, so it is likely that the structure could have been completed without experimental phase information (Fig. 6 ▶).

### 3.5. Comparison with standard refinement

For comparison, we performed ‘standard refinement’ consisting of three macrocycles of 200 steps of positional (*xyz*) minimization and 200 steps of restrained individual *B*-factor refinement using *CNS* starting from the same model that was used for DEN refinement. One round of automated model building starting from this standard refined model was performed using the same options for *AutoBuild* as for the DEN-refined model (see above).

The *R* values that were achieved by DEN refinement were significantly lower than those obtained by standard refinement (*e.g. R*_free_ = 0.444 *versus* 0.517; see Table 2 ▶). Moreover, the DEN-refined structure was significantly closer to the final model of Cgl1109 (representative examples are shown in Figs. 4 ▶ *a*, 4*c* ▶, 5 ▶ *a* and 5 ▶ *c*). Automated model building did not significantly improve the model after standard refinement (Figs. 4 ▶ *b* and 5 ▶ *b*; Table 2 ▶), resulting in *R*_free_ = 0.483 compared with *R*_free_ = 0.418 for the DEN-refined model. This example demonstrates that DEN refinement produces significantly better models than standard refinement for starting models that are far from the true structure, enabling further improvements by automated model building with *AutoBuild*. In most places there was reasonable agreement between the final model and the 2*mF*_o_ − *DF*_c_ electron-density maps computed after DEN refinement or subsequent automated model building (Figs. 4 ▶ *f* and 5 ▶ *f*). In contrast, the electron-density maps obtained by standard refinement with and without subsequent automated model building were fragmented and exhibited incorrect connectivity in several places (Figs. 4 ▶ *e* and 5 ▶ *e*). Thus, structure completion would have been very difficult to achieve with manual model building and standard refinement.

### 3.6. Determination of selenium sites and MAD phasing

The model obtained from the first round of DEN refinement and automated model building was used to calculate anomalous difference Fourier maps at the peak wavelength (λ_3_). These difference maps produced difference peaks for the six selenium sites of the SeMet residues in the protein. The positions of these six sites closely matched the positions of the Se atoms in the model obtained after DEN refinement and automated model building. Fig. 7 ▶ shows the standard deviations from the mean of the map (σ) of these six sites and the highest noise peak. The standard deviations of the peaks are compared with those obtained from standard refinement with and without subsequent automated model building. The combination of DEN refinement and automated model building produced the most significant difference peaks, all of which were well separated from noise. Standard refinement produced the poorest results, with three of the sites close to noise peaks. For both standard refinement and DEN refinement automated model building with *AutoBuild* improved the significance of the sites, although DEN refinement alone still produced more significant peaks for some of the sites than standard refinement and automated model building. In retrospect, it may have been possible to obtain the positions of the six sites by *ab initio* search, for example by using the *HySS* submodule (Grosse-Kunstleve & Adams, 2003 ▶), although careful choice of the high-resolution limit is required (truncation to 4.5 Å resolution) since a search against all diffraction data produced only one site that matched one of the six selenium sites.

We next calculated MAD phase probability distributions to 2.97 Å resolution and refined the six selenium sites using a maximum-likelihood method (Burling *et al.*, 1996 ▶) as implemented in *CNS* (Brünger *et al.*, 1998 ▶) using the mad_phase.inp task file. The diffraction data collected at the three wavelengths were used (Table 1 ▶), anisotropic scale factors between the three data sets were refined, individual *B* factors for the anomalous sites were refined, occupancies were set to 1 and anomalous form factors were constrained to be identical for all sites at a particular wavelength. The phasing calculations resulted in an overall figure of merit of 0.55 with reasonable overall scale factors, *B* factors and anomalous form factors of *f*′ = −6.14 (−6.95), *f*′′ = 4.73 (3.15) at the peak, *f*′ = −11, *f*′′ = 5.27 at the inflection point and *f*′ = −3.32 (−3.59), *f*′′ = 3.66 (1.05) at the remote wavelength, where the numbers refer to the results from the Friedel mate *F* to *F*_reference_ lack-of-closure expressions and the numbers in parentheses refer to the *F* to *F*_reference_ lack-of-closure expressions (Burling *et al.*, 1996 ▶). For comparison, the predicted values obtained from a fluorescence scan of the crystal are *f*′ = −8.65, *f*′′ = 6.21 at the peak, *f*′ = −11.11, *f*′′ = 3.64 at the inflection point and *f*′= −1.70, *f*′′ = 3.30 at the remote wavelength. In our experience, the differences between the refined values of *f*′ and *f*′′ for the two lack-of-closure expressions and from the predicted values are not uncommon for SeMet MAD data.

The resulting MAD electron-density map was subjected to density modification as implemented in *CNS* (Brünger *et al.*, 1998 ▶) using the density_modify.inp task file. The default settings were used, which include solvent flipping with generation of the mask based on root-mean-square electron-density fluctuations assuming 70% solvent content. No atomic model was used for the generation of the mask and no prior phase information was used for the refinement of anomalous sites in order to avoid model bias. The resulting figure of merit was 0.81 and the density-modified MAD electron-density map was connected but did not allow unambiguous identification of side chains for many residues (Figs. 4 ▶ *g* and 5 ▶ *g*). Although this map may be of sufficient quality such that manual building could have been attempted, it would have been challenging at this resolution. Indeed, automated model building using the same map resulted in a very incomplete model: only 76 side chains were fitted out of 360, with several false backbone connections.

### 3.7. Semi-automated completion of the refinement

A second round of DEN refinement (using the current model obtained from the first round of DEN refinement and automated model building as both the starting and the reference model) and automated model building was performed using the MLHL target function (Pannu *et al.*, 1998 ▶) that included the experimental MAD phase information, resulting in relatively small localized changes in coordinates with some more significant corrections of side-chain positions, improvements in *R* values and a reduction of the *R*_free_ − *R*_cryst_ difference (Table 2 ▶).

As mentioned above, there were several regions that required correction of register shifts and rebuilding of α-­helices (a particular example is shown in Fig. 6 ▶) that were not corrected even in the second round of DEN refinement and automated model building. To correct these regions, selected regions were deleted from the model and another round of automated rebuilding with *AutoBuild* was performed, again using the electron-density map from the previous model as the initial map, using the experimental MAD phase information and the primary sequence, with morphing enabled and the rebuild-in-place option set to false. Interestingly, we found that using a 2*mF*_o_ − *DF*_c_ electron-density map as the initial electron-density map for *AutoBuild* produced somewhat better results for rebuilding in this particular case than using the density-modified map generated by *AutoBuild*. The resulting models (using models with different deletions as starting models for automated model building) were inspected using *Coot* (Emsley *et al.*, 2010 ▶) and the portions that best fitted the electron-density maps were combined to generate a hybrid model. Missing loops were fitted with the ‘Fit Loops’ feature of *PHENIX*. This procedure of selected rebuilding by deletion of the problematic regions and automated rebuilding was repeated several times. This semi-automated method corrected the majority of cases of incorrectly fitted α-helices and loops arising from register errors (Figs. 4 ▶ *d* and 5 ▶ *d*, yellow *versus* orange models).

The remaining misfitted regions were manually corrected with *Coot* (Emsley *et al.*, 2010 ▶) interspersed with refinement with *phenix.refine* (Adams *et al.*, 2010 ▶). The final refinement (Table 1 ▶) employed residues 10–369 of Cgl1109 (a 369-residue protein) and other solvent molecules (one phosphate ion and one chloride ion). It was performed against diffraction data collected at the high-energy remote wavelength (Table 1 ▶).

### 3.8. Biological implications and comparison between 1vgy and Cgl1109

*C. glutamicum* is a Gram-positive bacterium that finds industrial use in the production of vitamins and amino acids, including glutamic acid, which is used in the production of the flavoring agent monosodium glutamate. Cgl1109 (NCBI reference sequence identifier NP_600337; UniProt identifier Q59284) is a putative succinyl-diaminopimelate desuccinylase (DapE) from *C. glutamicum* consisting of two domains: a peptidase domain belonging to family PF01546 (Peptidase_M20) in clan CL0035 of zinc metallopeptidases (∼30 000 proteins in 12 families) in v.25 of the Pfam database (Finn *et al.*, 2010 ▶) and a dimerization domain belonging to PF07687 (M20_dimer). These proteins have a broad phylogenetic spread across all kingdoms of life, show substantial sequence divergence and are essential for numerous biological processes (for example, recombinant bacterial carboxy­peptidase G2 is used in cancer therapy to hydrolyze methotrexate and is being tested in prodrug therapy, and human aspartoacylase is implicated in Canavan’s disease in the brain), but structural coverage exists for only a small fraction (∼0.3%) of the proteins in this clan. Cgl1109 was selected by the JCSG to increase the structural coverage of these families and is one of ∼20 structures determined to date (see http://www.topsan.org/Groups/Zinc_Peptidase). DapE is involved in producing l-lysine and l,l-2,6-diaminopimelate and its catalytic mechanism is likely to involve two zinc ions.

The crystal structure of Cgl1109 reveals a dimeric structure from crystal-packing considerations and as also suggested by the *PDBePISA* server (Fig. 2 ▶). The dimeric assembly is promoted by the smaller of the two domains of the molecule (Fig. 8 ▶), while the larger domain is the putative catalytic domain. The dimeric assembly is consistent with proteins from this family that contain a similar dimerization domain. Electron density in 2*mF*_o_ − *DF*_c_ and *mF*_o_ − *DF*_c_ maps initially suggested the possible presence of two zinc ions in the putative catalytic site, which would be expected owing to the addition of 6 m*M* ZnCl_2_ during cocrystallization (which was added based on putative functional annotation and ligand screening in a fluorescence-based thermal shift assay). However, we did not model zinc ions in the final model owing to the uncertainty associated with high *B* factors and the absence of significant peaks in the anomalous difference Fourier maps, including from diffraction data collected at the zinc absorption edge.

Fig. 8 ▶ shows a superposition of Cgl1109 with the template used for homology modeling (PDB entry 1vgy, chain *A*), a putative succinyl-diaminopimelate desuccinylase from *N. meningitidis*. The superposition shows that the overall fold is identical, but that there are large differences in secondary-structural element placement and length, as perhaps expected considering the low sequence identity (25%) between the proteins and the resulting difficulties with molecular-replacement phasing.

## 4. Conclusions

**Figure 5:**
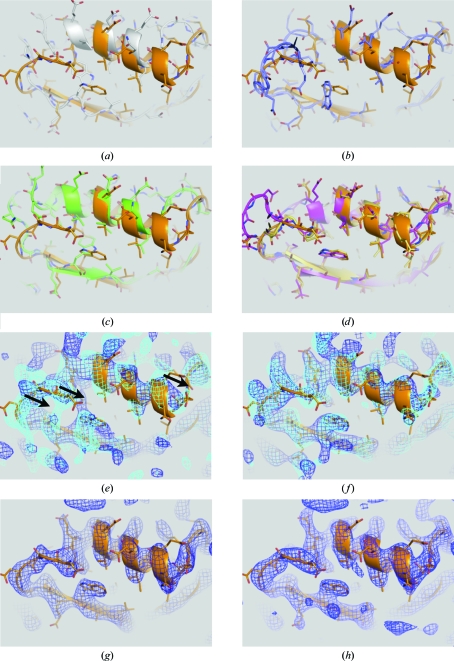
Comparison of various refinements and maps for residues 251–276. Residues 251–263 comprising an α-helix, residues 264–271 comprising a loop and residues 272–276 comprising a β-strand are shown (the sequence numbers refer to the genomic sequence; see Fig. 1 ▶). (*a*) Standard refinement (gray) *versus* the final model (orange). Standard refinement produces fragmented or incorrectly connected electron density (marked by arrows). (*b*) Standard refinement and one round of *AutoBuild* (blue) *versus* the final model (orange). Electron density is still fragmented or shows incorrect connectivity. (*c*) DEN refinement (green) *versus* the final model (orange). (*d*) DEN refinement and one round of *AutoBuild* (magenta) *versus* the result of semi-automated rebuilding (yellow). (*e*) 2*mF*_o_ − *DF*_c_ electron-density map after standard refinement (blue mesh) and a subsequent round of *AutoBuild* (cyan mesh) *versus* the final structure (orange sticks). (*f*) 2*mF*_o_ − *DF*_c_ electron-density map after DEN refinement (blue) and a subsequent round of *AutoBuil*d (cyan) *versus* the final structure (orange sticks). (*g*) Electron-density map obtained by density modification of the MAD map (blue) *versus* the final structure (orange sticks). (*h*) 2*mF*_o_ − *DF*_c_ electron-density map (blue mesh) of the final model (orange sticks).

Successful structure determination of the difficult molecular-replacement example Cgl1109 illustrates the synergism between DEN refinement and automated model building with *AutoBuild*. DEN refinement is most beneficial at the early stage of the refinement process, immediately after molecular-replacement phasing, when the model is still relatively crude and distant from the true structure. For Cgl1109, DEN refinement resulted in a model that is closer to the true structure, producing improved model phases that in turn provide a better starting point for automated model building. The improved model phases also provided more significant peaks in anomalous difference Fourier maps to better locate the six selenium sites of the protein. In contrast, standard refinement (*i.e.* positional and *B*-factor refinement) produced fragmented electron density with incorrect connectivity (marked by arrows in Fig. 5 ▶ *e*). The *R* values that we obtained after the initial round of DEN refinement and automated model building with *AutoBuild* are better than those reported in Table 1 of DiMaio *et al.* (2011 ▶) (*R*_free_ = 0.418 *versus* *R*_free_ = 0.460). This difference is most likely to arise from performing a full (γ, *w*_DEN_) grid search with multiple repeats with different initial velocities and random selection of DEN restraints at each grid point in the present work as opposed to a single DEN refinement as was performed previously (DiMaio *et al.*, 2011 ▶). Our success in fully refining the Cgl1109 structure also demonstrates that the combination of DEN refinement and automated model building is a viable alternative to the *Rosetta* molecular-replacement approach (DiMaio *et al.*, 2011 ▶). However, further analysis is required to determine the optimal application and potential limitations of both methods.

**Figure 6:**
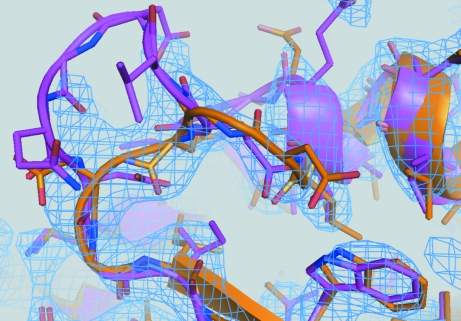
Comparison of various refinements and maps for residues 251–276. A close-up view of the loop consisting of residues 264–271, which is also part of Fig. 5 ▶, is shown. The final model is colored orange (sticks and cartoon representation). The structure after the first round of DEN refinement and *AutoBuild* is colored magenta (sticks and cartoon representation) and the corresponding 2*mF*_o_ − *DF*_c_ electron-density map (with model phases calculated from this structure, but without experimental phase information, and contoured at 1.4σ) is colored marine blue. The electron-density map clearly shows that the loop needed to be corrected.

**Figure 7:**
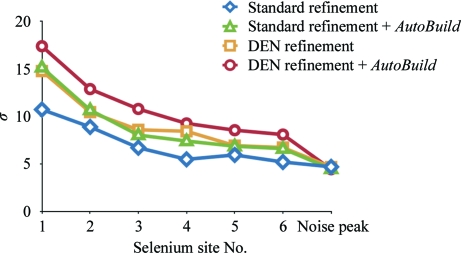
Significance of selenium sites. The standard deviation above the mean (σ) in anomalous difference Fourier maps is shown for the six selenium sites of the SeMet variant of Cgl1109. For comparison, the standard deviation of the highest noise peak is also shown. The amplitudes for the calculation of the anomalous difference Fourier map were obtained from the diffraction data at the peak wavelength (Table 1 ▶). The phases were obtained from the atomic model after standard refinement (blue diamonds), standard refinement followed by automated building with *AutoBuild* (green triangles), DEN refinement (yellow squares) and DEN refinement followed by automated model building with *AutoBuild* (red circles).

**Figure 8:**
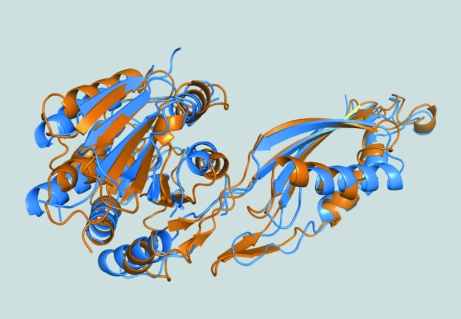
Comparison of Cgl1109 with 1vgy-*A*. A superposition of the final model of Cgl1109 (orange cartoon) and chain *A* of PDB entry 1vgy (blue cartoon) is shown. The superposition was performed with *PyMOL* (DeLano, 2002 ▶).

Poorly fitted portions of the model after DEN refinement and automated model building were readily identified by inspection of the electron-density maps (Fig. 6 ▶). These electron-density maps unambiguously suggested how to correct the model. It turned out that most of these regions were related to local sequence misalignments. We generated a structure-based alignment between the template 1vgy-*A* and Cgl1109 using *MUSTANG* (Konagurthu *et al.*, 2006 ▶) and compared it with predicted alignments. *PROMALS*3*D* and *HHpred* correctly assigned 282 and 291 positions (of a total of 360 residues visible in the Cgl1109 structure), respectively. The difference between the *PROMALS*3*D* and *HHpred* alignments is caused by a one-register shift involving an α-helix (residues 132–140). This one-residue shift required manual rebuilding when using the *PROMALS*3*D* alignment for the molecular-replacement search model. In retrospect, it might have been beneficial to use models generated by both the *PROMALS*3*D* and *HHpred* alignments as starting points for DEN refinement and automated model building and then to generate a composite model keeping the best-fitting parts of both models.

Sequence-register errors that arise from local misalignments between the target protein and the homology model can be difficult to correct using automated model-building methods when working with electron-density maps at low resolution or those based on highly anisotropic diffraction data. Overinterpretation or misinterpretation of such low-resolution maps is a real danger when they are manually interpreted without assistance from more objective computational methods. Indeed, we were able to partially automate the process by deleting the incorrectly aligned regions and rebuilding the parts with automated methods; some remaining regions had to be manually corrected. In particular, *AutoBuild* will sometimes misfit α-helices at low resolution, tracing the chain through the center of the α-helix (Fig. 5 ▶ *d*, magenta). It should be noted, however, that in this case the method of deleting the α-helix from the current model and rebuilding it from scratch produced the correct fit (Fig. 5 ▶ *d*, yellow). However, in two other instances this approach was not successful and the α-helices had to be manually rebuilt. It seems possible that this process could be fully automated. This would be especially important for low-resolution structures, in which interpretation of the electron-density map by inspection can be subjective and can lead to local misfitting (DeLaBarre & Brunger, 2005 ▶; Davies *et al.*, 2008 ▶). It is conceivable that a systematic method to probe the fit with different local sequence alignments in problematic regions might produce the best possible model for such low-resolution structures.

## Supplementary Material

PDB reference: succinyl-diaminopimelate desuccinylase, 3tx8

## Figures and Tables

**Table 1:** Crystallographic data and refinement statistics for Cgl1109 Values in parentheses are for the highest resolution shell.

	λ_1_ MAD-Se (remote)	λ_2_ MAD-Se (inflection point)	λ_3_ MAD-Se (peak)
Space group	*P*6_5_22		
Unit-cell parameters (Å)	*a* = 82.90, *b* = 82.90, *c* = 364.18		
Data collection			
Wavelength (Å)	0.9116	0.9794	0.9792
Resolution range (Å)	29.5–2.97 (3.05–2.97)	29.5–3.17 (3.26–3.17)	29.5–2.97 (3.05–2.97)
No. of observations	73623	60577	111259
No. of unique reflections	16179	13404	16192
Completeness (%)	99.1 (98.5)	99.1 (99.0)	99.2 (98.4)
Mean *I*/σ(*I*)	13.1 (1.5)	14.9 (2.8)	17.2 (1.7)
*R*_merge_ on *I*† (%)	9.5 (124.8)	9.1 (64.7)	10.6 (150.6)
*R*_meas_ on *I*‡ (%)	10.1 (140.6)	10.4 (72.9)	11.4 (162.5)
Model and refinement statistics			
Resolution range (Å)	29.5–2.97		
No. of reflections (total)	16098§		
No. of reflections (test set)	1649		
Completeness (%)	99.07		
Data set used in refinement	λ_1_ MAD-Se		
Cutoff criterion	|*F*| > 0		
*R*_cryst_¶	0.238		
*R*_free_¶	0.257		
Stereochemical parameters			
Restraints (r.m.s.d. observed)			
Bond angles (°)	0.625		
Bond lengths (Å)	0.003		
Average protein isotropic *B* factor (Å^2^)	99.7††		
Maximum-likelihood-based coordinate error (Å)	0.71		
Protein residues	360		
Phosphates/chlorides	1/1		

† *R*_merge_ = 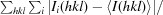 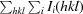 (Diederichs & Karplus, 1997 ▶)

‡ *R*_meas_ (redundancy-independent *R*_merge_) = 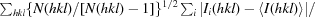 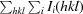.

§ Typically, the number of unique reflections used in refinement is slightly less than the total number that were integrated and scaled. Reflections are excluded owing to negative intensities and rounding errors in the resolution limits and unit-cell parameters.

¶ *R*_cryst_ = 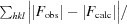 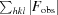, where *F*_calc_ and *F*_obs_ are the calculated and observed structure-factor amplitudes, respectively. *R*_free_ is the same as *R*_cryst_, but calculated using 10.24% of the total reflections that were chosen at random and omitted from refinement.

†† This value represents the total *B*, which includes overall TLS refinement and residual *B* components.

**Table 2:** *R* values for different refinement stages and, for comparison, for standard refinement

Structure	*R*_free_	*R*_cryst_
*Phaser* solution	—	0.649
Standard refinement	0.517	0.432
Standard refinement + *AutoBuild*	0.483	0.374
DEN refinement	0.444	0.399
DEN refinement + *AutoBuild*	0.418	0.327
Second DEN refinement (MLHL)	0.397	0.366
Second DEN refinement + *AutoBuild* (MLHL)	0.372	0.325
Final refined	0.257	0.238
